# Effect of smoking on coronavirus disease susceptibility: A case-control study

**DOI:** 10.18332/tid/156855

**Published:** 2023-01-25

**Authors:** Abdulelah Almansour, Naela B. Alamoudi, Sarah AlUrifan, Sundus Alarifi, Jumana Alagil, Rahmah M. Alamrie, Abdullatif Althunyan, Abdullah Alghumlas, Abdullah Alreedy, Abdulaziz Farea, Shaher Alshehri, Arwa Alumran

**Affiliations:** 1 Family and Community Medicine Department, College of Medicine, Imam Abdulrahman Bin Faisal University, Dammam, Saudi Arabia; 2 Emergency Medicine Department, King Fahad Specialist Hospital, Eastern Health Cluster, Ministry of Health, Dammam, Eastern Province, Saudi Arabia; 3 Dental Department, Ministry of Health, Eastern Province, Saudi Arabia; 4 General Dentist, Private Dental Practice, Khobar, Eastern Province, Saudi Arabia; 5 General Practitioner, Ministry of Health, Eastern Province, Saudi Arabia; 6 Department of Health Information Management and Technology, College of Public Health, Imam Abdulrahman bin Faisal University, Dammam, Saudi Arabia

**Keywords:** smoking, cigarettes, COVID-19, susceptibility, quarantine

## Abstract

**INTRODUCTION:**

Tobacco use has changed since the onset of the coronavirus disease (COVID-19) pandemic. The effect of smoking on COVID-19 susceptibility has not yet been determined. In this study, we aimed to assess the association between smoking and COVID-19 susceptibility.

**METHODS:**

This retrospective case-control study was conducted at the quarantine center of Imam Abdulrahman Bin Faisal University, Dammam, Saudi Arabia, between April and June 2020. A total of 142 adults participated in the study, 73 of whom tested positive for COVID-19 and were matched for both sex and age with participants in the control group. Telephone interviews were conducted to assess the risk factors associated with that exposure.

**RESULTS:**

Different variables are investigated for their impact on COVID-19 infection susceptibility. The current study’s findings indicated that smokers comprised only 27.5% (n=39) of the participants. There was no association between the COVID-19 swab results and smoking status (χ^2^=1.857; p=0.395). Furthermore, there was no significant association between any of the smoking parameters and susceptibility to COVID-19, except for the smoking period (t= -2.105, p=0.041). The odds of having a positive swab result among cigarette smokers were lower than those among pipe, waterpipe, and electronic cigarette smokers (OR=0.600; p=0.394). An association was also observed between COVID-19-positive swab results and contact with an individual with COVID-19 or respiratory disease (χ^2^=79.270 and χ^2^=18.929, respectively, p≤0.001).

**CONCLUSIONS:**

This study revealed no association between smoking status and COVID-19 swab test results. Further research with a bigger sample size is suggested to confirm the relationship between smoking and COVID-19 susceptibility.

## INTRODUCTION

The coronavirus disease (COVID-19) pandemic has had a significant effect on individual lifestyles, worldwide^1^. It has restricted people’s outings as a management measure during the pandemic and has led to an increase in the implementation of strict hygiene measures. The emergence of this pandemic has motivated a large proportion of the population to take better care of their health and protect themselves against infections. Nonetheless, this forced some individuals to start or increase their smoking habits even though it could be an opportunity to quit smoking because of the restrictive measures implemented. These findings can be attributed to either increased stress levels or boredom elicited by quarantine confinement^2^.

Severe acute respiratory syndrome coronavirus 2 (SARS-CoV-2), the virus responsible for COVID-19, uses the angiotensin-converting enzyme (ACE)-2 as a receptor to enter the cells. The association between the renin–angiotensin–aldosterone system and COVID-19 infection is unclear. Several systematic reviews and meta-analyses have concluded that patients with a smoking history are at higher risk of severe COVID-19^3-7^. In fact, the risk is increased by approximately two times for smokers compared to former smokers or non-smokers^7^. Smoking is a significant contributor to cardiovascular and respiratory disease^8^. This can be explained by the fact that smoking worsens both viral and bacterial infections by inducing structural and mechanical alterations in the respiratory system, and by inducing cell- and humoral-mediated immune responses^9,10^.

Since COVID-19 is an upper respiratory tract illness, we assume smoking habits may make people more susceptible to being infected. Our objective was thus to assess the impact of smoking on the COVID-19 susceptibility of infected quarantined persons and compare it with healthy persons in all quarantine centers run by Imam Abdulrahman Bin Faisal University (IAU) in the Eastern Region of Saudi Arabia.

## METHODS

This retrospective matched case-control study included adult individuals isolated at the IAU quarantine centers in Dammam and Alkhobar, Saudi Arabia, between April and June 2020. This study included both Saudi and non-Saudi Arabian men and women aged >18 years with known COVID-19 test results done by Polymerase Chain Reaction testing through the nasopharynx.

Participants’ data were obtained from the medical data of IAU quarantine’s database using a randomized sampling procedure, which was developed by the Department of Health Information Technology for quarantine use. Individuals who tested positive for COVID-19 were matched for both age and gender with participants who tested negative. The interviews were conducted by telephone to obtain more information about the demographics, history of contact factors, general health status two weeks before admission, and smoking status. All participants provided written informed consent upon admission for the study’s use of their information. Verbal consent was obtained from all participants during the telephone interview before it was initiated. The study’s details were given to the participants, including withdrawal instructions and assurance of confidentiality.

A total of 1846 individuals were quarantined, of those 150 tested positive for COVID-19 and were matched with 166 healthy persons who tested negative; the latter constituted the control group. This study aimed to assess the risk of infection associated with smoking status. We excluded the individuals with missing information or invalid COVID-19 test results. Only 73 of the 150 cases and 69 controls responded and agreed to participate. The IAU’s Institutional Review Board gave approval for this study (IRB-UGS- 2021-01-154) on 6 March 2021 and the study conforms with the Declaration of Helsinki.

Twenty-five patients were initially interviewed to evaluate inter-examiner reliability with a Cohen’s kappa value of at least 0.7, and were counted as part of sample size in the end. The information acquired from the respondents included demographics, such as age, sex, marital status, nationality, employment status, education level, body mass index (BMI, kg/m^2^), results of COVID-19 test, reason for quarantine, contact with individuals with COVID-19 or any other respiratory disease, and health/smoking status. Smoking status questions asked about whether a person was a current or ex-smoker in addition to questions regarding smoking type, age at initiation, duration, and reason for smoking. The Arabic version of the questionnaire was translated and back-translated into English, and the two versions were compared to assess translational validity.

The t-test and chi-squared tests were used for data analysis to compare between the study groups (positive- and negative-COVID-19 test results) in terms of sociodemographics and smoking status. All data were analyzed using SPSS/STATA version 27^11^.

## RESULTS

The descriptive analysis included 142 participants who volunteered to participate. Almost half of the participants had COVID-19, with a positive swab test result (n=73; 51.4%) (Table 1). This study included men (n=107; 75.4%), most of the participants were Saudi Arabians (n=130; 91.5%), and half of them were married (n=78; 54.9%). In terms of education level, 35.9% (n=51) were high school graduates, while 30.3% (n=43) held a Bachelor’s degree. Employed participants comprised 62.7% of the sample (n=89). The BMI ranged 18.5–24.9 kg/m^2^ (n=51; 35.9%). The average age was 33.46 ± 11.16 years.

**Table 1:** Demographic characteristics of study participants at the quarantine center of IAU, Dammam, Saudi Arabia, April–June 2020 (N=142)

*Characteristics*	*Total (N=142) n (%)*	*Swab results*		*Statistical test (p)*
		*Positive (N=73) n (%)*	*Negative (N=69) n (%)*	
**Age** (years), mean ± SD	33.46 ± 11.16	32 ± 11	35 ± 11	t= -1.151 (0.252)
**Sex**				χ^2^=0.603 (0.437)
Female	35 (24.6)	16 (21.9)	19 (27.5)	
Male	107 (75.4)	57 (78.1)	50 (72.5)	
**Nationality**				χ^2^=12.389 (<0.001)*
Saudi Arabian	130 (91.5)	61 (83.6)	69 (100)	
Other	12 (8.5)	12 (16.4)	0 (0)	
**Marital status**				χ^2^=3.978 (0.169)
Married	78 (54.9)	37 (50.7)	41 (59.4)	
Single	61 (43.0)	35 (47.9)	26 (37.7)	
Divorced	2 (1.4)	0 (0)	2 (2.9)	
Widowed	1 (0.7)	1 (1.4)	0 (0)	
**Education level**				χ^2^=9.877 (0.040)*
Intermediate school or lower	9 (6.3)	4 (5.5)	5 (7.2)	
High school	51 (35.9)	31 (42.5)	20 (29.0)	
Diploma	16 (11.3)	11 (15.1)	5 (7.2)	
Bachelor’s degree	43 (30.3)	21 (28.8)	22 (31.9)	
Postgraduate	23 (16.2)	6 (8.2)	17 (24.6)	
**Employment status**				χ^2^=5.295 (0.143)
Unemployed	31 (21.8)	15 (20.5)	16 (23.2)	
Employed	89 (62.7)	51 (69.9)	38 (55.1)	
Student	16 (11.3)	6 (8.2)	10 (14.5)	
Retired	6 (4.2)	1 (1.4)	5 (7.2)	
**BMI** (kg/m^2^)^a^				χ^2^=4.820 (0.191)
<18.5	4 (2.8)	3 (4.3)	1 (1.4)	
18.5–24.9	51 (35.9)	26 (37.1)	25 (36.2)	
25–29.9	42 (29.6)	16 (22.9)	26 (37.7)	
≥30	42 (29.6)	25 (35.7)	17 (24.6)	
**Reason for quarantine**				χ^2^=93.573 (<0.001)*
Contracted COVID-19 inside SA	55 (38.7)	54 (74.0)	1 (1.4)	
International arrival	85 (59.9)	18 (24.7)	67 (97.1)	
Companion with a COVID-19-positive person	2 (1.4)	1 (1.4)	1 (1.4)	
**Contact with a COVID-19-positive person**				χ^2^=79.270 (<0.001)*
No	106 (74.6)	32 (43.8)	4 (5.8)	
Yes	36 (25.4)	41 (56.2)	65 (94.2)	
**Contact with any respiratory disease**				χ^2^=18.929 (<0.001)*
No	112 (78.9)	26 (35.6)	4 (5.8)	
Yes	30 (21.1)	47 (64.4)	65 (94.2)	
**Health status**				χ^2^=13.898 (0.008)*
Very bad	2 (1.4)	2 (2.7)	0 (0)	
Bad	2 (1.4)	2 (2.7)	0 (0)	
Acceptable	3 (2.1)	3 (4.1)	0 (0)	
Good	18 (12.7)	14 (19.2)	4 (5.8)	
Very good	117 (82.4)	52 (71.2)	65 (94.2)	

a Three values are missing.

* Significant results.

The main reason for being quarantined was international arrival (n=85; 59.9%), while the least common reason was contact with COVID-19-positive individuals (n=2; 1.4%). Among the participants, 74.6% reported not having any type of contact with a COVID-19-positive person in the two weeks prior to their quarantine (n=106), while 25.4% reported that they had (n=36). In addition, 78.9% reported not having contact with a person with respiratory symptoms two weeks prior to quarantine (n=112), while 21.2% reported that they had (n=30). Many respondents (n=117; 82.4%) reported having ‘very good’ health status, and only four participants (2.8%) reported having either ‘very bad’ or ‘bad’ health status (Table 1).

More than half of our study participants were non-smokers (n=96; 67.6%), and current smokers comprised only 27.5% (n=39) (Supplementary file Table 1). Among those who were current smokers or ex-smokers, half smoked cigarettes (n=26; 56.5%), while only 4.3% (n=2) smoked electronic cigarettes and almost one-third smoked waterpipes (n=12; 26.1%).

The average age at which the participants started smoking was 21 ± 6 years, and the average period of smoking was 11 ± 8 years, from the onset of smoking until the commencement time of the study (Supplementary file Table 1). Furthermore, most smokers indicated that they had started smoking as a test (n=29; 63%), 26% of them were imitating their family or friends (n=12), while 5 (10.9%) because of stress or to increase their self-confidence.

There was no association between COVID-19 swab results and smoking status (χ^2^=1.857; p=0.395) (Supplementary file Figure 1), smoking type (χ^2^=0.730; p=0.552), age at the onset of smoking (t= -0.363; p=0.718), or the reason for the smoking initiation (χ^2^=2.313; p=0.378). However, the period of smoking significantly influenced swab results (t= -2.105; p=0.041) (Supplementary file Figure 2). The shorter the smoking period, the higher the likelihood that a participant had a positive COVID-19 swab result (Supplementary file Table 2).

Furthermore, the crude odds ratio revealed no significant association between any of the smoking parameters and susceptibility to COVID-19. However, the odds of having a positive result if the reason for smoking was stress or increased self-confidence, was three times higher than that of copying smokers from the family or friends or testing (p=0.318). Additionally, cigarette smokers had lower odds of having a positive swab result for COVID-19 than pipe, waterpipe, or electronic cigarette smokers (OR=0.600; p=0.394).

Further analysis showed that there was an association between COVID-19 swab results and nationality (χ^2^=12.389; p=0.001), education level (χ^2^=9.877; p=0.040), quarantine (χ^2^=93.573; p=0.001), contact with an individual with COVID-19 (χ^2^=79.270; p=0.001) or an individual who had a respiratory disease (χ^2^=18.929; p=0.001), and health status (χ^2^=13.898; p=0.008) (Table 1). Other demographic characteristics, including sex, marital status, employment status, BMI, and age, were not significantly associated with increased susceptibility to COVID-19.

## DISCUSSION

This study examined the relationship between smoking status and likelihood of contracting COVID-19. Other demographic factors were examined to determine whether they were linked to COVID-19. Almost one-third of the study participants were smokers. This was greater than the proportion of current smokers (14%) reported by the Centers for Disease Control and Prevention in 2019. However, this percentage declined from 20.9% in 2005^12^. This is in accordance with the trends in the prevalence of tobacco smoking reported by the World Health Organization regarding tobacco smoking since the beginning of the 21st century, indicating that smoking rates have been steadily decreasing^13^. In addition, smoking was more common among men than women (15.3% and 12.7%, respectively). It was highest among people aged 25–44 years (16.7%) and 45–64 years (17%). Furthermore, people with general education level certificates constituted the highest number (35.3%), whereas those with a graduate degree constituted the lowest number (4%)^12^. A study conducted in Saudi Arabia in 2010 reported that the proportion of smokers ranged from 11.6% to 34.4%. This wide range may be the result of sampling techniques that do not represent the entire population^14^.

Smoking has been implicated in several respiratory diseases, including infections^15^. The inflammatory reaction caused by smoke exposure leads to impairment of lung function and inflammation in pulmonary epithelial cells. This activates TGF-β in the epithelial airway, leading to the release of pro-inflammatory mediators by alveolar macrophages to facilitate inflammation and fibrosis^16,17^. Therefore, smoking has an adverse effect on the immune response of the respiratory system^18^. Moreover, cigarette smoke can cause further destruction of the alveolar wall and lead to mucus hypersecretion by inducing the production of inflammatory mediators including IL-1β, IL-8, TNF-α, and IFN-γ. This exaggerated immune response leads to the formation of reactive oxygen and reactive nitrogen species^19,20^. In addition, with the action of proteolytic enzymes, pulmonary tissue becomes even more damaged^21^. Evidence has shown a significantly increased risk of Middle East respiratory syndrome (MERS)-related mortality in smokers compared with non-smokers^22^. As MERS-CoV and SARS-CoV-2 belong to the same Coronaviridae family, it can be deduced that smoking is a risk factor for COVID-19^23-24^.

The differences between this study’s findings and those mentioned above may be attributed to various limitations and confounders. Some studies that have assessed the relationship between COVID-19 and smoking have focused on hospitalized patients, thus imposing a limitation on communicating with patients in severe and critical conditions. Additionally, socioeconomic status was considered a confounder in these studies because hospital access is unequally available to people from different socioeconomic backgrounds. In addition, smoking has respiratory and cardiovascular consequences that may overlap with COVID-19 complications^10^.

Our study reported a significant correlation between education level and COVID-19 contraction. According to a recent study, people with a higher education level are more likely to participate in healthy behaviours^25^, whereas participants with lower education level believe that they are less susceptible to contracting COVID-19 and are less aware of the risk it implies, and consequently have a higher vulnerability to contracting COVID-19^26^.

Regarding the history of contact with infected people, we found that it was significantly correlated with positive COVID-19 swab results, irrespective of the presence of symptoms. This supports that COVID-19 is transmitted through contact and respiratory transmission^27-31^. However, these findings differ from those reported by Tian et al.^32^ and Chen et al.^33^, in which such a correlation was not found.

In addition, this study found no significant relationship between COVID-19 infection and BMI. This result is in accordance with that of a prospective cohort study involving 89 adolescents, which reported no significant relationship between BMI and cardiorespiratory fitness^34^. In contrast, obesity is an independent risk factor for contracting COVID-19 as it has a significant impact on disease susceptibility and severity^34^.

### Limitations

This study has a few limitations, including unintentional operator bias, where some answers were subjective to the respondents, and recall bias during the smoking questionnaire, which could have significantly affected the results. The sample size was another limitation; the population of this study was restricted to IAU quarantine residents, resulting in a relatively small sample size that might compromise the generalizability of the results. In addition, even though this study found a correlation between smoking and COVID-19, there is no established causal reason for this relationship.

## CONCLUSIONS

This research showed no significant association between smoking status and the COVID-19 susceptibility. Other variables, such as contact history with a COVID-19-positive individual and education level, were associated with increased susceptibility to contracting COVID-19. BMI did not significantly influence susceptibility to COVID-19. It is important to note that the current results are applied to positive cases with moderate to low risk, excluding hospitalized COVID-19 cases. Further investigations, including systematic reviews, meta-analyses, and larger sample size studies, are needed to draw firm conclusions regarding the relationship between smoking and COVID-19 susceptibility.

## Data Availability

The data supporting this research are available from the authors on reasonable request.
