# Supplementary material for: Effect of smoking on coronavirus disease susceptibility: A case-control study
Source: Tob Induc Dis. 2023 Jan 25;21:11. doi: 10.18332/tid/156855 (PMC9875716; doi:10.18332/tid/156855)
Supplement: Supplementary file 1 [file TID-21-11-s1.pdf]

Supplementary Materials

Tables

Table 1. Influence of smoking on the susceptibility of COVID-19 at the quarantine centre of IAU, Dammam, Saudi Arabia between April and June 2020 (*n*=142).

Variable	Frequency (%)	COVID-19		Test (*P*-value)
		Positive *n*= 73 (%)	Negative *n*= 69 (%)	
**Smoking status**				
-Non-Smoker	96 (67.6)	49 (67.1)	47 (68.1)	x^2=1.857
-Current Smoker	39 (27.5)	22 (30.1)	17 (24.6)	(.40)
-Ex-Smoker	7 (4.9)	2 (2.7)	5 (7.2)	
**Type of smoking**				
-Cigarette	26 (56.5)	15 (62.5)	11 (50.0)	x^2=.730
-Pipe, Water pipe, or electronic cigarettes	20 (43.5)	9 (37.5)	11 (50.0)	(.55)
**How did you start smoking?**				
-Stress or 'to increase self-confidence'	5 (10.9)	3 (12.5)	2 (9.1)	x^2=2.313
-Imitation of a family member/ friend	12 (26.1)	4 (16.7)	8 (36.4)	(.38)
-Test	29 (63.0)	17 (70.8)	12 (54.5)	
**Smoking starting age** (mean ± SD)	20.78 ± 6	20.5 (5.9)	21.1 (6.8)	*t*=-.363 (.72)
**Period of smoking** (mean ± SD)	10.69 ± 8	8.3 (6.6)	13.4 (9.4)	*t*=-2.105 **(.04)***

* Significant results

Table 2. The odds, crude and the adjusted odds ratio of the influence of smoking on the susceptibility of COVID-19

**Variable**	**Crude**		**Adjusted**	
	**OR (*P*-Value)**	**95% CI**	**OR (P-Value)**	**95% CI**
**Smoking status**				
-Non-Smoker	.38 (.27)	.07, 2.08	-[a]	-[a]
-Current Smoker	.31 (.19)	.05, 1.80		
-Ex-Smoker	1	-		
**Type of smoking**				
-Cigarette	.60 (.39)	.19, 1.94	1.02 (.97)	.27, 3.91
-Pipe, Water pipe, or electronic cigarettes	1	-	1	-
**How did you start smoking?**				
-Stress or 'to increase self-confidence'	.94 (.05)	.14, 6.55	1.12 (.92)	.14, 8.95
-Imitation of a family member/ friend	2.83 (.15)	.69, 11.60	2.02 (.38)	.42, 9.68
-Test	1	-	1	-
**Smoking starting age** (mean ± SD)	1.02 (.71)	.93, 1.12	1.04 (.45)	.93, 1.17
**Period of smoking** (mean ± SD)	1.08 (.05)	1.00, 1.18	1.09 (.07)	.99, 1.19

[*] Significant results
[a] Smoking status was not included in the adjusted odds ratio model, as only smokers or ex-smokers were included.

Figure legends

Figure 1. Association between smoking status and COVID-19 infection at the quarantine centre of IAU, Dammam, Saudi Arabia between April and June 2020 (n=142)

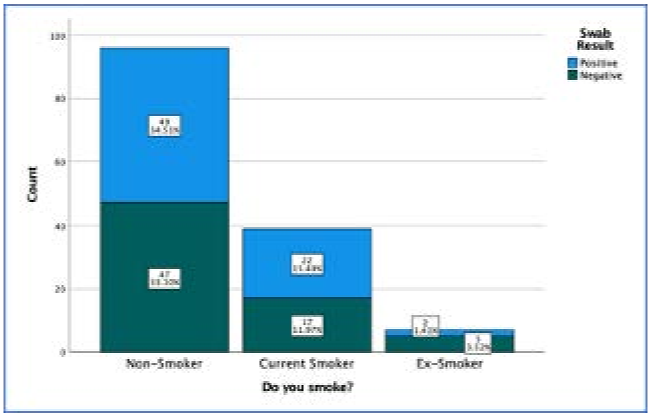

Figure 2. Association between smoking period and COVID-19 infection at the quarantine centre of IAU, Dammam, Saudi Arabia between April and June 2020 (n=142)

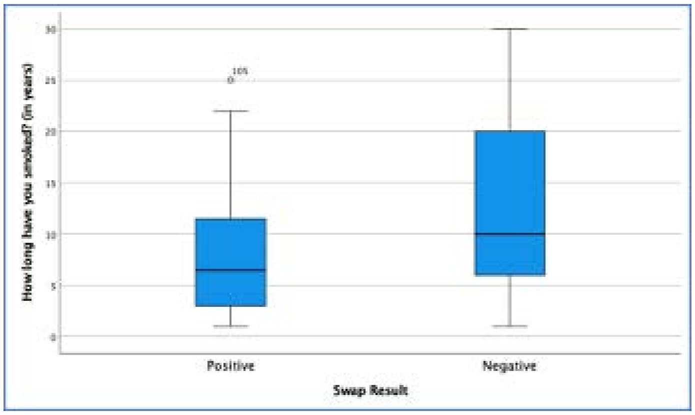

© 2023 Almansour A. et al.
